# Genetic Polymorphisms within The Intronless *ACTL7A* and *ACTL7B* Genes Encoding Spermatogenesis-Specific Actin-Like Proteins in Japanese Males

**DOI:** 10.22074/ijfs.2019.5702

**Published:** 2019-07-14

**Authors:** Hiromitsu Tanaka, Yasushi Miyagawa, Akira Tsujimura, Morimasa Wada

**Affiliations:** 1 Faculty of Pharmaceutical Sciences, Nagasaki International University, Huis Ten Bosch, Sasebo, Nagasaki, Japan; 2 Department of Urology, Graduate School of Medicine, Osaka University, Yamadaoka, Suita, Osaka, Japan; 3 Department of Urology, Juntendo University Hospital, Hongo, bunnkyouku, Tokyo, Japan

**Keywords:** Germ Cell, Infertility, Single Nucleotide Polymorphism, Sperm, Testis

## Abstract

Actins play essential roles in cellular morphogenesis. In mice, the T-actin1 and 2 genes, which encode actin-like proteins, are specifically expressed in haploid germ cells. Both *T-ACTIN1/ACTLB* and *T-ACTIN2/ACTL7A* have also been cloned and studied. The orthologous genes in humans are present on chromosome 9q31.3 as intronless genes. Defects of germ cell-specific genes can introduce infertility without somatic function impairment. We determined T- ACTIN1 and 2, specifically expressed in the testis using reverse-transcription polymerase chain reaction (RT-PCR). To examine whether genetic polymorphisms of the *T-ACTIN1* and 2 genes are associated with male infertility, we screened for *T-ACTIN1* and 2 polymorphisms by direct sequencing of DNA from 282 sterile and 89 fertile Japanese men. We identified five and six single nucleotide polymorphisms (SNPs) in the *T-ACTIN1* and 2 regions of the sterile and fertile subjects respectively. Among these genetic polymorphisms was a novel SNP that was not in the National Center for Biotechnology Information SNP database. Although we could not determine whether these SNPs cause infertility, the prevalence of these genetic polymorphisms may be useful for analyzing polymorphisms in future large- scale genetic analyses.

After meiosis, round spermatids undergo a dramatic change to develop the specific morphology of the mature sperm. Actin proteins play important functions in this process (1). We developed a mouse subtracted library including genes specifically expressed in spermatogenesis and cloned and characterized these genes (2). Among these genes were *T-actin1* and 2, which encode actin-like proteins and are specifically expressed in haploid germ cells. T-actin1 is located in the cytoplasm while T-actin2 is localized in the nuclei of testicular haploid germ cells and is present only in the heads and tails of sperm (3). In both the mouse and human genome, *T-ACTIN1* and 2 are positioned head-to-head and lack introns (4, 5). The resulting amino acid sequences, genomic construction, and cAMP response elements (CRE) consensus DNA sequence of the promoters of *T-actin1* and 2 are conserved in mice (4). These genes have been reported to cause infertility by inducing autoimmunity to sperm (6). Human *T-ACTINs* may play important roles in the specific morphogenesis of spermatozoa during spermiogenesis, as well as in sperm function.

We investigated genetic polymorphisms in the DNA sequences of germ cell-specific genes in infertile male patients and male volunteers with confirmed fertility (7- 19) to identify polymorphisms potentially linked to male infertility (7, 8, 15, 19). In this study, we report our analysis of genetic polymorphisms in *T-ACTIN1/ACTLB* and *T-ACTIN2/ACTL7A* in Japanese men.

Defects in germ cell-specific genes may be a cause of idiopathic infertility. To detect the presence of small amounts of transcripts, we examined tissue-specific expression patterns of *T-ACTIN1* and 2 by reverse-transcription polymerase chain reaction (RT-PCR) using cDNA from various organs and a Rapid-Scan gene expression panel containing cDNA from different human tissues (OriGene Technologies, Rockville, MD, USA) (20). The specific primers:

TACT1-RTF: 5'-ATGGCGACAAGGAACAGCCCCATG-3'
TACT1-RTR: 5'-TCAGCACTTGCTGTAGATGGCCAC-3'
  for *T-ACTIN1*
TACT2-RTF: 5'-ATGTGGGCTCCACCAGCAGCAATC-3'
TACT2-RTR: 5'-TCAGAAGCACCTTCTGTAGAGGAAG-3'
  for *T-ACTIN2*

were designed to amplify fragments from the open reading frames. Polymerase chain reaction (PCR) was performed using Gflex Hot Start (Takara, Japan). The cycling conditions were 96°C for 2 minutes, followed by 35 cycles of denaturation at 96°C for 45 seconds, annealing at 58°C for 45 seconds, and extension at 68°C for 90 seconds. As a control, *ß-actin* was also amplified using primers:

ACTBF: 5'-ACCGAGGCCCCCCTGAACCC-3'
ACTBR: 5'-TCCATCATGAAGTGTGACGT-3'

according to the manufacturer’s protocol. *T-ACTINs* were specifically detected only in the testis (Fig .1). 

**Fig 1:**
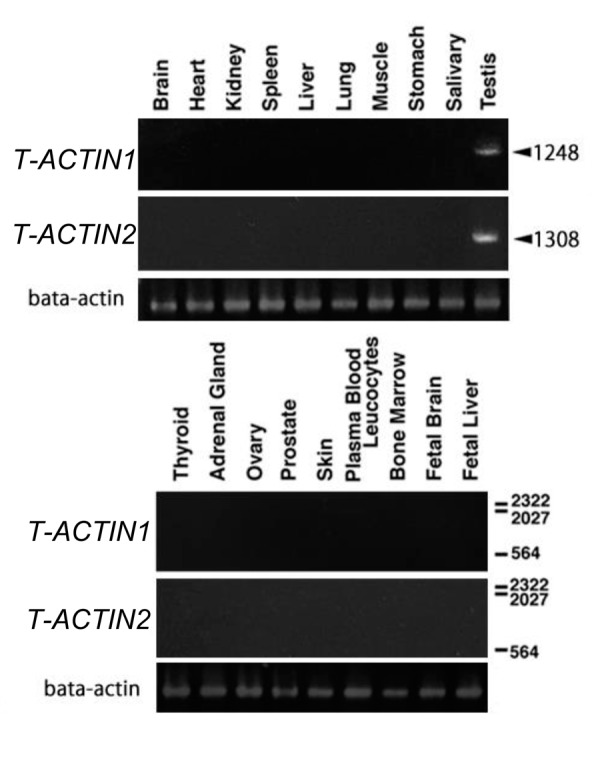
mRNA expression of *T-ACTIN1* and 2 in various human organs. Multiple human tissue cDNAs were subjected to polymerase chain reaction analysis. Fragments of *T-ACTIN1* and 2 were specifically detected in the testes. Numbers in the right-hand margin indicate the lengths of the amplified fragments and DNA ladder makers. The expression of actin mRNA was also examined as a control.

The entire coding sequences of *T-ACTIN1* and 2 (National Center for Biotechnology Information [NCBI] accession number: chromosome 9, NC_000009.12 (108862228..108863755), Fig.2) are intronless, similar to mouse T-actin1 and 2. As *T-ACTINs* are expressed at high levels in the human testis (Fig .1), we investigated whether genetic polymorphisms in *T-ACTINs* are associated with male infertility.

**Fig 2:**
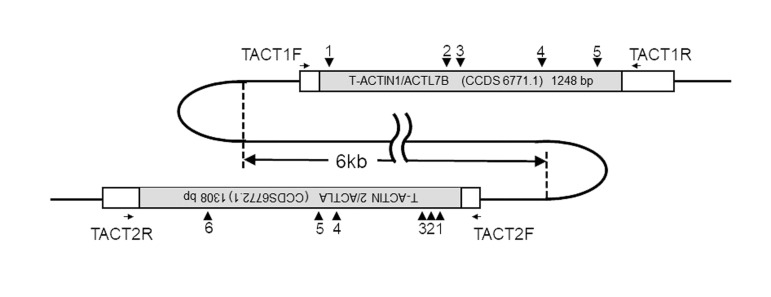
Schematic view of the *T-ACTIN1* and 2 genes. The *T-ACTIN1* and 2 intronless genes are located on chromosome 9 (NCBI accession number: NC_000009.12). The box indicates the transcribed region of the *T-ACTIN1* and 2 genes. The open reading frame is shaded. *T-ACTIN1* is transcribed to the right and *T-ACTIN2* to the left. The small horizontal arrows in the box indicate the locations of the polymerase chain reaction (PCR) and DNA-sequencing primers. The arrowheads indicate the positions of genetic polymorphisms. The NCBI accession numbers of *T-ACTIN1* and 2 are CCDS4295.1 and CCDS6772.1, respectively.

Infertile Japanese subjects (n=282) were divided into subgroups according to the degree of defective spermatogenesis: 192 patients (68%) had non-obstructive azoospermia, and 90 (32%) had severe oligospermia (<5×10^6^ cells/mL), according to the criteria of the World Health Organization (Table 1). All patients had idiopathic infertility based on cytogenetic analysis and no history of other medical conditions, including cryptorchidism, recurrent infections, trauma, orchitis, varicocele, and others. The control group consisted of fertile males who had fathered children born at a maternity clinic (n=89). All donors were informed of the purpose of the study and gave permission for use of their blood for genomic DNA data. This study was approved by the institutional review board and independent ethics committee of Osaka University.

**Table 1:** Backgrounds of 371 Japanese men


Status	n (%)

Azoospermia	192 (68)
Severe oligospermia	90 (32)
Total infertile	282 (100)
Fertile control	89


Genomic DNA was isolated from blood samples by protease treatment and phenol extraction. *T-ACTIN1* and 2 sequences were amplified through PCR using the following primers:

TACT1F: 5'-GTGGATCCCTGGATGGTCCGCTGTGCGG- 3'
TACT1R: 5'-GGCCTGTGCCATCTGTGCTGGAGG-3'

for *T-ACTIN1*,

TACT2F: 5'-CTTTCAGGCCTTGAATCCAGTGGG-3' 

TACT2R: 5'-GGTAGGCACTGCCAGTGCAGTGTC-3' for T-ACTIN 2 (Fig .2). 

PCR was performed using Ex Taq Hot Start (Takara, Japan) and consisted of 40 cycles of 96ºC for 45 seconds, 65ºC for 45 seconds, and 72ºC for 90 seconds. PCR-amplified fragments were purified using SUPREC PCR spin columns (Takara). The resulting DNA fragments were sequenced independently from both ends by the same PCR protocol using thermal cycle sequencing kits (Applied Biosystems, Foster City, CA, USA). Internal primers:

TACT1F2: 5'-GCCTGTGCCATCTGTGCTGG-3'
TACT2F2: 5'-TCTCAAGCTGGTTAACCCTCTGCG-3'
TACT2R2: 5'-AGGCACTGCCAGTGCAGTGT-3'

were used to confirm *T-ACTIN* genes with ambiguous identifications. The reaction products were analyzed using an ABI-PRISM 310 Genetic Analyzer (Applied Biosystems). Differences in variables between the experimental and control conditions were compared using Fisher’s exact test (P<0.05).

Nucleic acid base exchanges introducing one nonsense mutation and four silent mutations were found in the *T-ACTIN1* open reading frame (Table 2). Single nucleotide polymorphisms (SNPs) were found in three silent mutations (48C>T, 561C>T, 870C>T) as minor genotypes in the entire Japanese cohort. The minor 1137 C>T homozygous alleles on *T-ACTIN1* was not detected in the infertile group. One nonsense mutation was found in the volunteer group. The translated region of *T-ACTIN1* is 1248 bp long, and the nonsense mutation appears at base pair 1,171, near the C-terminus. This mutation thus has little influence on the function of the translated protein, making it unlikely to be a cause of infertility.

**Table 2:** Prevalence of single nucleotide polymorphisms (SNPs) in *T-ACTIN1* in infertile or proven fertile populations


	Position			Genotype	Number (%) of SNP				Reference	
	Nucleotide^*^	Amino acid			Infertile (%)		Proven fertile (%)		(NCBI dbSNP rs#)	

*T-ACTIN1/ACTL7B*	48	16	D	C/C	161 (57.1)		54 (60.7)		rs3750468	
				C/T	102 (36.2)		28 (31.5)			
				T/T	19 (6.7)		7 (7.9)			P=0.74
	561	187	Y	C/C	161 (57.1)	54 (60.7)		rs11543179		
				C/T	102 (36.2)		28 (31.5)			
				T/T	19 (6.7)		7 (7.9)			P=0.74
	870	290	T	C/C	218 (77.7)	66 (74.2)		rs3750467		
				C/T	62 (22.0)		21 (23.6)			
				T/T	2 (0.7)		2 (2.2)			P=0.23
	1137	379	S	C/C	282 (100)	87 (97.8)		rs769443334		
				C/T	0 (0)		2 (2.2)			
				T/T	0 (0)		0 (0)			
	1171	391	Q	C/C	282 (100)	88 (98.9)		rs750564969		
			Q/Ter	C/T	0 (0)		1 (1.1)			
			Ter	T/T	0 (0)		0 (0)			
Total					282		89			


D; Aspartate, Y; Tyrosine, T; Threonine,S; Serine, Q; Glutamine, Ter; Termination, and *; The nucleotide positions relative to the first methionine.

**Table 3:** Prevalence of single nucleotide polymorphisms (SNPs) in *T-ACTIN1* in infertile or proven fertile populations


	Position			Genotype	Number (%) of SNP		Reference
	Nucleotide^*^	Amino acid			Infertile (%)	Proven fertile (%)	(NCBI dbSNP rs#)

*T-ACTIN1/ACTL7B*	118	40	R	C/C	28 (99.6)	89 (100)	rs201549336
				C/A	1 (0.4)	0 (0)	
				A/A	0 (0)	0 (0)	
	133	45	R	C/C	282 (100)	88 (98.9)	rs368653764
			R/C	C/T	0 (0)	1 (1.1)	
			C	T/T	0 (0)	0 (0)	
	153	51	P	A/A	218 (99.6)	89 (100)	In present study
				A/	1 (0.4)	0 (0)	
				G/G	0 (0)	0 (0)	
	528	176	P	A/A	278 (98.6)	88 (98.9)	rs3739692
				A/T	4 (4.1)	1 (1.1)	
				T/T	0 (0)	0 (0)	
	657	219	V	G/G	261 (92.6)	82 (92.1)	rs3739693
				G/A	21 (7.4)	4 (4.5)	
				AA	0 (0)	3 (3.4)	
	1018	340	V	G/G	261 (92.6)	82 (92.1)	rs7872077
			V/M	G/A	21 (7.4)	4 (4.5)	
			M	A/A	0 (0)	3 (3.4)	
Total					282	89	


R; Arginine, C; Cysteine, P; Proline, V; Proline, M; Methionine, and *; The nucleotide positions relative to the first methionine.

Two nucleic acid base exchanges introducing amino acid substitutions and four silent mutations were found in the *T-ACTIN2* open reading frame (Table 3). The frequency of minor genotypes associated with *T-ACTIN2* nucleotide polymorphisms was low in Japanese males. One silent mutation, 153A>G, in *T-ACTIN2* was not registered in the NCBI SNP database (dbSNP), marking a novel discovery in our Japanese cohort. 

Logistic regression modeling of the prevalence of haplotypes, including SNPs, revealed no significant differences between major and minor alleles lacking the 1,018 G>A on *T-ACTIN2* SNP in males proven to be fertile. The minor 1,018 G>A homozygous alleles on *T-ACTIN2* in males proven to be fertile is considered to be due to an error made by the sequencer.

The appearance of 48 C>T and 561 C>T in *T-ACTIN1* was linked; as was the appearance of 657 G>A and 1,018 G>A in *T-ACTIN2*. Thus, the SNPs in these two genes may have the same origin.

Although many SNPs have been registered in the NCBI dbSNP, we detected only 11 genetic polymorphisms in the open reading frames of the *T-ACTIN* genes among 371 Japanese men. Finally, z χ^2^-test was used to compare genotype distributions between infertile males and proven fertile controls. There were no significant differences for the minor genotypes (P>0.05).

Our research group has focused on cloning and analyzing germ cell-specific genes. Chromosome mapping of these genes revealed that they are distributed across various chromosomes, and that many are intronless (21). *T-ACTINs* are among these intronless genes and are specifically expressed in the testis (Fig .2). The dysfunction of germ cell-specific genes does not affect ontogeny and may be a cause of unexplained male infertility. The dysfunction of these genes in mice has been shown to lead to infertility (22). Dominant-negative gene mutations are not passed on to the next generation, however other gene mutations can be inherited from a heterozygous male parent or from the female parent. More than 20% of married couples in Japan are affected by infertility and the male partner is responsible in two-thirds of these cases (23). We undertook an extensive analysis of genetic polymorphisms in germ cell-specific genes and of the relationship between gene polymorphisms and infertility (7-19). We found potential relationships between infertility in Japanese men and genetic polymorphisms or mutations in *PRM2, TP1, PGAM4,* and *SCOT-T* (7, 8, 15, 19). We analyzed SNPs in germ cell-specific genes and found that some included genetic polymorphisms with single amino acid substitutions, whereas other specific genes had few genetic polymorphisms. Most genes having several genetic polymorphisms encoded in proteins were involved in signal transduction or regulation, whereas those with few genetic polymorphisms were more likely to encode structural proteins (12). In this study, we discovered several different SNPs in *T-ACTIN1* and 2 in a cohort of Japanese men. The similar frequencies of these polymorphisms between the fertile and infertile groups in this study imply that these mutations are not associated with male infertility. However, the prevalence data for these genetic polymorphisms might be useful when analyzing the association of traits and genetic polymorphisms in further large-scale genetic analyses.
